# First Insights into Clinical and Resistance Features of Infections by *Klebsiella pneumoniae* among Oncological Patients from a Referral Center in Amazon Region, Brazil

**DOI:** 10.3390/idr12030021

**Published:** 2020-12-03

**Authors:** Anderson Lineu Siqueira dos Santos, Yan Corrêa Rodrigues, Marcos Vinícios Hino de Melo, Pabllo Antonny Silva dos Santos, Tatyellen Natasha da Costa Oliveira, Daniele Melo Sardinha, Luana Nepomuceno Gondim Costa Lima, Danielle Murici Brasiliense, Karla Valéria Batista Lima

**Affiliations:** 1 Parasite Biology in the Amazon Region (PPGBPA), Center of Biological and Health Sciences, State University of Pará (UEPA), Belém 66087-662, Brazil; yan.13@hotmail.com (Y.C.R.); hinomarcos6@gmail.com (M.V.H.d.M.); antonnypabllo@gmail.com (P.A.S.d.S.); tatyellenoliveira@iec.gov.br (T.N.d.C.O.); luanalima@iec.gov.br (L.N.G.C.L.); 2 Epidemiology and Health Surveillance (PPGEVS), Evandro Chagas Institute (IEC), Ananindeua 67030-000, Brazil; danielle-vianna20@hotmail.com; 3 Bacteriology and Mycology Section, Evandro Chagas Institute (IEC), Ananindeua 67030-000, Brazil; daniellemurici@iec.gov.br

**Keywords:** *Klebsiella pneumoniae*, neoplasms, multi-drug resistance, Brazil

## Abstract

*Klebsiella pneumoniae* appears as one of the most prevalent pathogens among cancer patients. The present study investigates the clinical, epidemiological and microbiological aspects related to infections caused by *K. pneumoniae* in cancer patients treated at an oncology referral center in the state of Pará, Amazon region, Brazil. Between July 2017 to July 2019, an epidemiological, observational, cross-sectional study, with a descriptive and analytical approach was conducted, including patients with confirmed diagnosis of cancer who acquired infection by *K. pneumoniae* 72 h after hospital admission. *K. pneumoniae* isolates included in the study were obtained from different clinical materials (blood, urine, catheter tip and bladder catheter, orotracheal secretions, oncological and surgical wounds). Antimicrobial susceptibility testing and molecular detection of the carbapenemase-encoding genes were performed. A high prevalence of MDR *K. pneumoniae* isolates was observed, including two colistin-resistant isolates and seven isolates harboring bla_KPC-1_ gene. To conclude, our findings provide the firsts insights into the epidemiology and infection by *K. pneumoniae* in the state of Pará, Brazil, and may be useful on treatment guidance and establishment of strategies to control the spread of resistance strains of *K. pneumoniae* in the region.

## 1. Introduction

*Klebsiella pneumoniae* stands as one of the most prevalent pathogens among cancer patients and a major complication among those undergoing chemotherapy, with infection and mortality rates due to bacteremia ranging from 10% to 53% and 18% to 30%, respectively [1,2,3,4]. Worryingly, mortality rates reaching 72.7% have been reported due to the spread of multidrug-resistant (MDR) and carbapenemase-producing *K. pneumoniae* strains [5,6,7].

The prevention of healthcare-acquired infections (HAIs) based on evidence represents a challenge for health institutions, depending on contextual factors of healthcare systems and involving health promotion and patients’ safety [8]. The investigation of these factors is necessary and timely, as the prevalence of HAIs is higher in developing countries, especially in Brazil, where prevalence rates of HAIs reaches 22.8% compared to 9% in European countries [9]. Prolongations in antineoplastic treatment, dosage reduction of chemotherapeutic drugs, lengthy hospital stays, suboptimal therapeutic response and increase in hospital and outpatient costs are also associated with *K. pneumoniae* infection episodes [8,9].

Several factors are associated with increased risk of infections among cancer patients. Severe and prolonged neutropenia induced by chemotherapy is the main risk factor for the acquisition of serious infections, including those caused by MDR pathogens [10,11]. Moreover, immunocompromised individuals demonstrate few clinical signs in bacterial infections, which are commonly confused with the symptoms of the underlying neoplasm or treatment side effects [7,10,11]. Other risk factors that contribute to poor prognosis infections in cancer patients are myeloablative therapy, total body irradiation, high doses of steroids, tissue grafting, prolonged hospitalization, recent surgery, resection of multiple organs, exposed cancer, enteral diet, ostomies, admission to intensive care unit (ICUs), presence of central venous catheter, bladder catheterization, ventilatory assistance, hemodialysis and disease chronic pulmonary disease [12,13,14,15].

Recently, the increasing resistance to carbapenems and limited options for antimicrobial therapy led to the reinsertion of polymyxins into clinical practice [16,17]. Although associated with high nephrotoxicity and neurotoxicity, polymyxins are currently one of the last-resort treatment drugs for MDR *K. pneumoniae* [18].

The aim of this study was to investigate the clinical, epidemiological and resistance aspects related to infections caused by *K. pneumoniae* among cancer patients treated at an oncology referral center in the state of Pará, Amazon region, Brazil.

## 2. Materials and Methods

### 2.1. Study Setting, Patients Data and Inclusion Criteria

This is an epidemiological, observational, cross-sectional study, with a descriptive and analytical approach, conducted at a tertiary referral public hospital in chronic-degenerative diseases, which functions as the center of high complexity in oncology and the main reference in cancer treatment in the public network of the state of Pará, Brazil. The hospital has 236 beds, 29 of which in ICUs.

Patients were included in the study according to the following criteria: (I) patients with confirmed diagnosis of cancer, regardless of topography, with or without metastasis and who acquired infection by *K. pneumoniae* 72 h after hospital admission; (II) admitted between July 2017 to July 2019 and (III) over 18 years of age. Patients with different pathologies unrelated to neoplasms and with infection associated with other microorganisms were excluded.

Clinical and epidemiological data were collected from patients’ medical records. The following independent variables were considered: sex, age, type of cancer, treatment, cancer wound, ostomy, use of catheters, length of admission, diet before infection, presence of comorbidity, infection site, presence of bacteremia, antimicrobial treatment and clinical outcome.

### 2.2. Bacterial Isolates and Antimicrobial Susceptibility Testing

*K. pneumoniae* isolates included in the study were obtained from different biological sources, including: blood, urine, catheter tip and bladder catheter, orotracheal secretions, oncological and surgical wounds. Bacterial suspensions for each sample were prepared to match the 0.5 McFarland standard, followed by bacterial identification and antimicrobial susceptibility testing on the *Vitek-2 System (bioMérieux, Marcy l’Etoile, France*) using *Vitek-2* card AST 239 for the following antibiotics: amikacin (AMI); ampicillin (AMP); ampicillin + sulbactam (SAM); cefepime (FEP); cefoxin (FOX); ceftazidime (CAZ); ceftriaxone (CRO); ceftriaxone acetyl (CXA); cefuroxime (CXM); ciprofloxacin (CIP); colistin (CS); ertapenem (ETP); gentamicin (GM); imipenem (IMP); meropenem (MEM); piperacillin + tazobactam (TZP) and tigecycline (TGC). As results, isolates were classified as sensitive (S), intermediate (I) and resistant (R), and the results were interpreted according to the Clinical and Laboratory Standards Institute (CLSI) criteria and breakpoints [19]. Reference strains *Escherichia coli* ATCC 25922 and *Pseudomonas aeruginosa* ATCC 27853 were used as quality control.

### 2.3. Phenotypical and Molecular Detection of Carbapenemase

All isolates non-susceptible to imipenem and/or meropenem were investigated for the presence of carbapenemase by phenotypic test of inactivation of carbapenem (mCIM/eCIM), in accordance with the CLSI recommendations [19]. The presence of carbapenemases-encoding genes (bla_KPC_, bla_NDM-like_, bla_IMP-like_, bla_VIM-like_ and bla_SPM-like_) was investigated by polymerase chain reaction (PCR) as previously described, and using primers presented in Table 1 [20,21].

### 2.4. Statistical Analyses

The collected information was grouped and processed using the Statistical Package for Social Science (SPSS), version 17.0. Antimicrobial susceptibility profile was defined as the dependent variable, with an inversion in the death variable. Fisher’s Exact tests or G-test of independence were used for comparative analysis between categorical variables; odds ratios (ORs) and the 95% confidence intervals (CIs) were estimated. *p*-values ≤ 0.05 were considered statistically significant.

### 2.5. Ethic Statement

The present study was conducted in accordance with Helsinki Declaration and with approval of the ethics and research Committee at Evandro Chagas Institute (IEC) (N°:1.915.939) and Ophir Loyola Hospital (HOL) (N°: 2.032.763).

## 3. Results

### 3.1. Clinical and Epidemiological Aspects

The present study included 64 oncologic patients, who developed infection by *K. pneumoniae* during admission period, of which 48.4% (31/64) were male and 51.6% (33/64) were female. The average age was 50.9 years, with higher prevalence of infection on the age group from 41 to 60 years’ old (46.9%—0/64). The most prevalent type of cancer was related with the reproductive system—ovary, cervix and prostate cancers (25.0%–16/64)—followed by kidney cancer (20.3%–13/64). Only 6.2% (4/64) of patients presented an oncological wound. As for the treatment of neoplasia, surgical intervention was the most performed procedure (35.9%–23/64), followed by the use of immunosuppressant drugs (34.4%–22/64) and chemotherapy (10.9%–7/64). Treatment procedure data were not available for 18.7% (12/64) of patients, who were excluded from the statistical analyses (Table 2).

Regarding the length of the hospital stay, most of the patients were hospitalized for less than 10 days when *K. pneumonia* infection was identified (59.4%, 38/64). Type of diet data revealed that 37.5% (24/64) of patients were on an enteral diet using a nasogastric or nasoenteric tube or a gastrostomy. Kidney (45.3%–29/64) and respiratory diseases (25.0%–16/97) were the most prevalent underlying diseases among patients (Table 2).

All patients presented the use of at least one invasive device. A total of 46.9% (30/64) of the patients presented the use of catheters, with a urinary catheter being the most frequent (29.7%–19/64). The central venous catheter was present in 50.0% (32/64) of the patients. Regarding the presence of ostomies, 39.5% (23/64) had some ostomy, with tracheostomy being the most prevalent (31.2%–20/64) (Table 2).

Infection site data were not available for 26 patients. Bloodstream infection (17.2%–11/64) and urinary tract infection (15.6%–10/64) were reported as the main infections sites among patients. A total of 84.4% (54/64) of the patients underwent antimicrobial treatment (Table 1). As for the outcome, 25% (16/64) of the participants died, of which 12.5% (2/16) were resistant to carbapenems and 81.2% MDR. Death of one patient was associated with a colistin-resistant isolate (Table 2).

### 3.2. Antimicrobial Susceptibility Features

Antimicrobial susceptibility testing revealed high prevalence of MDR *K. pneumoniae* isolates (49/64–77.0%), which were mainly resistant to ampicillin (95.3%), followed by cefuroxime (67.2%) and cefuroxime axetil (67.2%). Eleven isolates were not tested to colistin and 3.8% (2/53) showed resistance to the drug (Table 3). Seven isolates demonstrating positive results for mCIM and negative for eCIM were positive for the bla_KPC-1_ gene.

## 4. Discussion

Infections caused by carbapenemase-producing enterobacteria are increasingly being reported in patients in health institutions, which are more difficult to treat and control due to few or an absence of effective antimicrobials, leading to an increase in morbidity, mortality and hospital costs. Several health institutions consider *K. pneumoniae* to be a public health threat due to its highly tendency to acquire resistance mechanisms and present MDR or extra drug resistance (XDR) phenotypes, especially in hospital environment [22]. Thus, the present study provides novel and important data on the epidemiology of infections caused by *K. pneumoniae* in the Brazilian territory, highlighting the first report of high prevalence of MDR, emergence of resistance to colistin and *K. pneumoniae* isolates harboring the bla_KPC_ gene in the state of Pará, Brazil.

Recently, the occurrence of hypervirulent and MDR strains of *K. pneumoniae* has become a reason for concern in hospital environments, with the inherent characteristics of cancer patients favoring the spread of this pathogen [23,24]. Frequent prolonged antibiotic therapy interventions (on average 10 days) in these patients, due to neutropenia, seem to increase the rates of resistance to antimicrobials [25]. Lin et al. [26] emphasized that bacterial infections are the main clinical complication and are the leading causes of death in cancer patients, especially in developing countries. Other studies involving immunocompromised patients infected with *K. pneumoniae* MDR have reported high mortality, up to 78% in patients undergoing liver transplantation and 56% in patients with hematological neoplasms [27,28].

A study by Jo et al. [29] demonstrated that in cancer patients, antimicrobial resistance in *K. pneumoniae* is linked to misuse and/or overuse of chemotherapeutic agents. Due to a decreased immunity in individuals undergoing antineoplastic chemotherapy, opportunistic pathogens such as *K. pneumoniae* can cause a wide range of infections, including pneumonia, urinary tract infection, bacteremia and meningitis [30].

Kidd et al. [23] showed that infections by MDR microorganisms are associated with a longer hospital stay. However, the results of the present study show that the majority of occurrences of carbapenem-resistant *K. pneumoniae* infections occurred in the first 20 days of hospitalization, while in longer stays the frequency decreased. The same was identified for those patients infected by MDR isolates, who mostly had a hospital stay of less than 20 days. These findings differ from previous reports, however, highlighting the need to characterize populations from the difference hospital settings.

In spite of several reports of infections cause by *K. pneumoniae* strains, there are little data on the incidence and outcome of *K. pneumoniae* MDR infections in immunocompromised patients. In a study of 18 cases of bloodstream infection with carbapenem-resistant Enterobacteriaceae in patients with hematological malignancies, 14-day mortality was over 53% [27]. Freire et al. [5] identified a 48% mortality after 30 days of infection by MDR *K. pneumoniae*. The present study observed a mortality of 16 (25%) participants; however, there was no statistical relationship between the presence of resistance to carbapenems or MDR and the death outcome.

Worryingly, our data revealed the predominance of MDR phenotype among *K. pneumoniae* isolates in our population, including 17.1% of patients infected with carbapenem-resistant isolates. The European Disease Control and Prevention Center (ECDC), in 2017, warned of an increase in the number of cases of *K. pneumoniae* resistant to carbapenems, highlighting an increase in detection rates from 6.2% to 8.1% in an interval of just three years, from 2012 to 2015 [31]. As for resistance to colistin, similar results were listed by the same source, with a detection rate of 8.8% [31]. Currently, resistance to colistin has been increasingly reported in clinical isolates of *K. pneumoniae* worldwide [22,23,32,33,34,35,36]. Observing the polymyxin resistance profile and noting that colistin is considered the last treatment option for *K. pneumoniae*, the occurrence of two cases in this study with oncological patients signals attention with this group has also been the first report of colistin-resistant *K. pneumoniae* in a hospital setting in the state of Pará, Amazon region, Brazil. In addition, KPC carbapenemases is one of most relevant resistance-related mechanisms among *K. pneumoniae*. Our findings revealed the presence of seven isolates harboring the bla_KPC_ gene, demonstrating that this gene is widespread in several hospitals from different regions in Brazil [37,38,39]. Yet, there is still scarcity of data regarding the detection of bla_KPC_ gene in the Brazilian Amazon region, with only recent reports from Tocantins [38] and Amazonas [39] states, where the gene was detected among 25 and 5 isolates, respectively.

## 5. Conclusions

In conclusion, a high prevalence of MDR *K. pneumoniae* was observed in our study population, as well as a significant number of the occurrence of resistance to carbapenems, emergence of resistance to colistin and detection of bla_KPC_ gene in seven isolates. The obtained data highlight the need and urgency of strategies approaching the epidemiological surveillance of HAIs, combining the continuous work between the hospital infection control committees and healthcare workers. Moreover, a review of data regarding antimicrobial prescriptions, antimicrobial susceptibility tests results and the use of invasive devices in the hospital setting could provide better insights into the risk factors associated with *K. pneumonia* infections. Finally, our findings provide the firsts insights into the epidemiology and infection by *K. pneumoniae* in the state of Pará, Brazil, and may be useful on treatment guidance and establishment of strategies to control the spread of resistance strains of *K. pneumoniae* in the region.

## Figures and Tables

**Table 1:** Primers used for carbapenemase-encoding genes amplification.

Gene	Oligonucleotide Sequence (5′–3′)	Amplicon Size (bp)	Reference
*bla*_IMP_	F:GAATAG(A/G)(A/G)TGGCTTAA(C/T)TCTC	188	[20]
	R:CCAAAC(C/T)ACTA(G/C)GTTATC		
*bla*_VIM_	F:GTTTGGTCGCATATCGCAAC	382	
	R:AATGCGCAGCACCAGGATAG		
*bla*_SPM-1_	F:CTAAATCGAGAGCCCTGCTTG	798	
	R:CCTTTTCCGCGACCTTGATC		
*bla*_NDM_	F:GGTTTGGCGATCTGGTTTTC	621	[21]
	R:CGGAATGGCTCATCACGATC		
*bla*_KPC_	F:CGTCTAGTTCTGCTGTCTTG	798	
	R:CTTGTCATCCTTGTTAGGCG		

**Table 2:** Clinical and epidemiological aspects of cancer patients infected with *K. pneumoniae* according to the antimicrobial resistance profile.

	Carbapenems						Multidrug-Resistance					
	R		S				MDR		NOT MDR			
	n	%	n	%	P	OR (95% CI)	n	%	n	%	P	OR (95% CI)
**Sex**												
Male	4	6.3	27	42.2	0.512 *	0.55 (0.14–2.10)	25	39	6	9	0.5599 *	1.56 (0.48–5.06)
Female	7	10.9	26	40.6		-	24	38	9	14		-
**Age**												
18–40 years	4	6.3	12	18.8	0.620	1.95 (0.48–7.81)	11	17	5	8	0.7072	0.57 (0.16–2.05)
41–60 years	4	6.3	26	40.6		0.59 (0.15–2.26)	24	38	6	9		1.44 (0.44–4.66)
61–79 years	3	4.7	15	23.4		0.95 (0.22–4.07)	14	22	4	6		1.10 (0.29–4.04)
**Type of cancer**												
Hematological	0	0	3	4.7	0.420	-	3	4.7	0	0	0.8445	-
Soft parts	2	3.1	5	7.8		2.13 (0.35–12.74)	7	10.9	0	0		-
Digestive system	0	0	4	6.3		-	2	3.1	2	3.1		0.20 (0.02–1.71)
Reproductive system	4	6.3	12	18.8		1.95 (0.48–7.81)	11	17.2	5	7.8		0.35 (0.07–1.56)
Cranioencephalic	1	1.6	3	4.7		1.67 (0.15–17.70)	3	4.7	1	1.6		0.72 (0.06–7.94)
Renal	1	1.6	12	18.8		0.34 (0.03–2.94)	10	15.6	3	4.7		0.76 (0.16–3.68)
Not informed	3	4.7	14	21.9		-	13	20.3	4	6.3		-
**Oncological wound**												
Yes	1	1.6	3	4.7	0.986 *	1.67 (0.15–17.70)	46	71.9	14	21.9	0.9628 *	1.09 (0.10–11.38)
No	1	15.6	50	78.1		-	3	4.7	1	1.6		-
**Type of treatment**												
Chemotherapy	0	0	7	10.9	0.214	-	7	10.9	0	0	0.6046	-
Surgery	4	6.3	19	29.7		1.01 (0.24–4.29)	17	26.6	6	9.4		0.73 (0.20–2.69)
Immunosuppressants	5	7.8	17	26.6		1.91 (0.45–8.15)	16	25	6	9.4		0.64 (0.17–2.34)
Not informed	2	3.1	10	15.6		-	9	14.1	3	4.7		-
**Length of hospitalization (days)**												
<=10	7	10.9	31	48.4	0.515	1.43 (0.32–6.21)	28	46.7	10	16.7	0.3745	0.82 (0.24–2.82)
11–20	0	0	3	4.7		-	3	5	0	0		-
21–30	1	1.6	4	6.3		1.27 (0.13–12.80)	3	5	2	3.3		0.28 (0.04–1.99)
31–40	1	1.6	1	1.6		5.44 (0.31–95.21)	2	3.3	0	0		-
41–50	0	0	5	7.8		-	5	8.3	0	0		-
51+	1	1.6	6	9.4		0.81 (0.09–7.62)	4	6.7	3	5		0.22 (0.04–1.24)
**Diet before infection**												
Oral	8	12.5	30	46.9	0.505 *	1.87 (0.44–7.87)	28	43.8	10	15.6	0.5768	0.73 (0.21–2.49)
Enteral	3	4.7	21	32.8		-	19	29.7	5	7.8		-
Not informed	0	0	2	3.1		-	2	3.1	0	0		-
**Respiratory disease**												
No	8	12.5	40	62.5	0.997 *	-	35	54.7	13	20.3	0.3185 *	-
Yes	3	4.7	13	20.3		1.15 (0.27–5.00)	14	21.9	2	3.1		2.60 (0.51–13.04)
**Kidney disease**												
No	7	10.9	28	43.8	0.741 *	-	28	43.8	7	10.9	0.5595	-
Yes	4	6.3	25	39.1		0.64 (0.16–2.44)	21	32.8	8	12.5		0.65 (0.20–2.09)
Ostomy												
Yes	2	3.1	21	32.8	0.301 *	0.34 (0.07–1.72)	30	47	11	17	0.5418 *	0.57 (0.15–2.06)
No	9	14.1	32	50		-	19	30	4	6		-
**Type of ostomy**												
Cystostomy	1	1.6	3	4.7	0.986 *	1.67 (0.16–17.70)	4	17	0	0	0.567 *	-
Tracheostomy	2	3.1	18	28.1	0.479 *	0.43 (0.08–2.21)	16	67	4	17	0.759 *	1.33 (0.36–4.84)
Tubes												
Yes	4	6.3	26	40.6	0.520 *	0.59 (0.15–2.27)	25	39	9	14	0.5709	0.69 (0.21–2.24)
No	7	10.9	27	42.2		-	24	38	6	9		-
**Type of tube**												
Nasoenteric	2	3.1	11	17.2	1.000 *	0.84 (0.16–4.50)	11	17.2	4	6.2	0.736 *	0.79 (0.21–2.99)
Nasogastric	2	3.1	9	14.1	0.984 *	0.99 (0.18–5.37)	8	12.5	3	3.1	0.998 *	0.78 (0.21–3.41)
Bladder catheter	3	4.6	16	25.0	1.000 *	0.87 (0.20–3.70)	15	23.4	4	6.2	1.000 *	1.21 (0.33–4.43)
**Catheter**												
Peripheral Venous	6	9.4	26	40.6	0.755 *	1.25 (0.347–4.59)	23	36	9	14	0.3968 *	0.58 (0.18–1.91)
Central Venous	5	7.8	27	42.2		0.80 (0.22–2.95)	26	41	6	9		1.69 (0.52–5.49)
**Infection site**												
Surgery	0	0	2	3.1	0.612	-	1	2	1	2	0.5024	0.29 (0.01–4.96)
Central venous catheter	1	1.6	4	6.3		1.40 (0.13–15.26)	2	3	3	5		0.18 (0.02–1,22)
Not identified	0	0	2	3.1		-	1	2	1	2		0.29 (0.01–4.96)
Did not inform	5	7.8	21	32.8		-	20	31	6	9		-
Pulmonary	3	4.7	5	7.8		5.40 (0.84–34.80)	7	11	1	2		2.33 (0.26–20.65)
Blood	1	1.6	10	15.6		0.44 (0.04–4.27)	9	14	2	3		1.46 (0.27–7.65)
Urinary	1	1.6	9	14.1		0.51 (0.05–5.00)	9	14	1	2		3.15 (0.36–27.14)
**Bacteremia**												
No	4	6.3	15	23.4	0.626 *	-	15	23	4	6	1.000 *	-
Yes	1	1.6	10	15.6		0.37 (—/—)	9	14	2	3		-
Not informed	6	9.4	28	43.8		-	25	39	9	14		
Antimicrobial therapy												
No	0	0	6	9.4	0.577 *	-	5	8	1	2	1.000 *	-
Yes	9	14.1	45	70.3		-	41	64	13	20		0.63 (0.06–5.90)
**Death**												
No	7	10.9	31	48.4	0.709 *	1.58 (0.29–8.59)	28	43.8	10	15.6	0.732 *	-
Yes	2	3.1	14	21.9		-	13	20.3	3	4.7		1.55 (0.36–6.59)
Not informed	2	3.1	8	12.5		-	8	12.5	2	3.1		-

P = (*p*-value per Test G of independence). * P = (*p*-value by Fisher’s exact test).

**Table 3:** Antimicrobial susceptibility of *K. pneumoniae* isolated from oncological patients.

		Antimicrobial Susceptibility Testing									
Antimicrobial Class	Antimicrobial	S		R		I		NP		Total	
		n	%	n	%	n	%	n	%	n	%
Penicillin	AMP	0	0.0	61	95.3	3	4.7	0	0.0	64	100.0
	SAM	16	25.0	37	57.8	0	0.0	11	17.2	64	100.0
	TZP	31	48.4	24	37.5	9	14.1	0	0.0	64	100.0
	AMK	5	7.8	1	1.6	5	7.8	53	82.8	64	100.0
2nd generation cephalosporin	CXM	18	28.1	43	67.2	3	4.7	0	0.0	64	100.0
	CXA	18	28.1	43	67.2	3	4.7	0	0.0	64	100.0
	FOX	38	59.4	12	18.8	3	4.7	11	17.2	64	100.0
3rd generation cephalosporin	CAZ	24	37.5	26	40.6	3	4.7	11	17.2	64	100.0
	CRO	22	34.4	42	65.6	0	0.0	0	0.0	64	100.0
4th generation cephalosporin	FEP	49	76.6	15	23.4	0	0.0	0	0.0	64	100.0
Carbapenems	ETP	50	78.1	5	7.8	0	0.0	9	14.1	64	100.0
	IPM	42	65.6	10	15.6	1	1.6	11	17.2	64	100.0
	MEM	52	81.3	11	17.2	0	0.0	1	1.6	64	100.0
Aminoglycosides	AMK	64	100.0	0	0.0	0	0.0	0	0.0	64	100.0
	GEN	41	64.1	21	32.8	2	3.1	0	0.0	64	100.0
Quinolones	CIP	25	39.1	39	60.9	0	0.0	0	0.0	64	100.0
Glycylcyclines	TGC	41	64.1	10	15.6	2	3.1	11	17.2	64	100.0
Polymyxins	CST	51	79.7	2	3.1	0	0.0	11	17.2	64	100.0
Sulphonamides	SXT	3	4.7	8	12.5	0	0.0	53	82.8	64	100.0

S: sensitive; R: resistant; I: intermediate; NP: not performed.
